# A large adrenocortical adenoma surrounded with a renal arteriovenous malformation

**DOI:** 10.1002/iju5.12293

**Published:** 2021-05-03

**Authors:** Takato Nishino, Kazutaka Narimoto, Koki Tominaga, Masayuki Sano, Masaki Shimbo, Natsuka Muraishi, Naoki Kanomata, Kazunori Hattori

**Affiliations:** ^1^ Departments of Urology St. Luke’s International Hospital Chuo‐ku Tokyo Japan; ^2^ Departments of Radiology St. Luke’s International Hospital Chuo‐ku Tokyo Japan; ^3^ Departments of Pathology St. Luke’s International Hospital Chuo‐ku Tokyo Japan

**Keywords:** adrenocortical adenoma, hypervascularization, laparoscopic adrenalectomy, nidus, renal arteriovenous malformation

## Abstract

**Introduction:**

Large adrenal adenomas are clinically rare. We report a case of a large adrenal adenoma with a renal arteriovenous malformation, mimicking a malignant adrenal tumor in preoperative imaging.

**Case presentation:**

A 66‐year‐old woman presented to a local hospital with abdominal pain. A right adrenal tumor was detected, 66 mm in diameter and surrounded by thick and tortuous vessels. Based on the imaging findings, pheochromocytoma was suspected. However, clinical symptoms and endocrine abnormalities were absent, and radionuclide accumulation in scintigraphy was negative. Laparoscopic right adrenalectomy was performed. Intraoperatively, a notable growth of vessels forming a nidus surrounding the tumor was observed. Pathologically, this was diagnosed as an adrenocortical adenoma in conjunction with a renal arteriovenous malformation.

**Conclusion:**

We report a case of a large adrenal tumor surrounded with an arteriovenous malformation. To the best of our knowledge, this is the first reported case of this combination.

**Abbreviations & Acronyms**
ACTH: adrenocorticotropic hormone
ARR: aldosterone‐renin ratio
AVM: arteriovenous malformation
CT: computed tomography
HE: hematoxylin and eosin
HU: Hounsfield unit
MIBG: metaiodobenzylguanidine
MRI: magnetic resonance imaging
PAC: plasma aldosterone concentration
TSH: thyroid stimulating hormone

**Keynote message**

We report the first case of a giant adrenocortical adenoma coexisting with a renal AVM. Based on the imaging studies and pathological examination, we consider that the adrenal adenoma and the AVM occurred coincidentally.

## Introduction

Benign adrenal adenomas originating from the adrenal cortex with a maximal diameter > 5 cm are considerably rare clinically.^1^ We report a case of a giant adrenal adenoma with a renal AVM, mimicking a hypervascular malignant tumor. We report the first case and describe the imaging and surgical findings, with a literature review.

## Case presentation

**Fig. 1:**
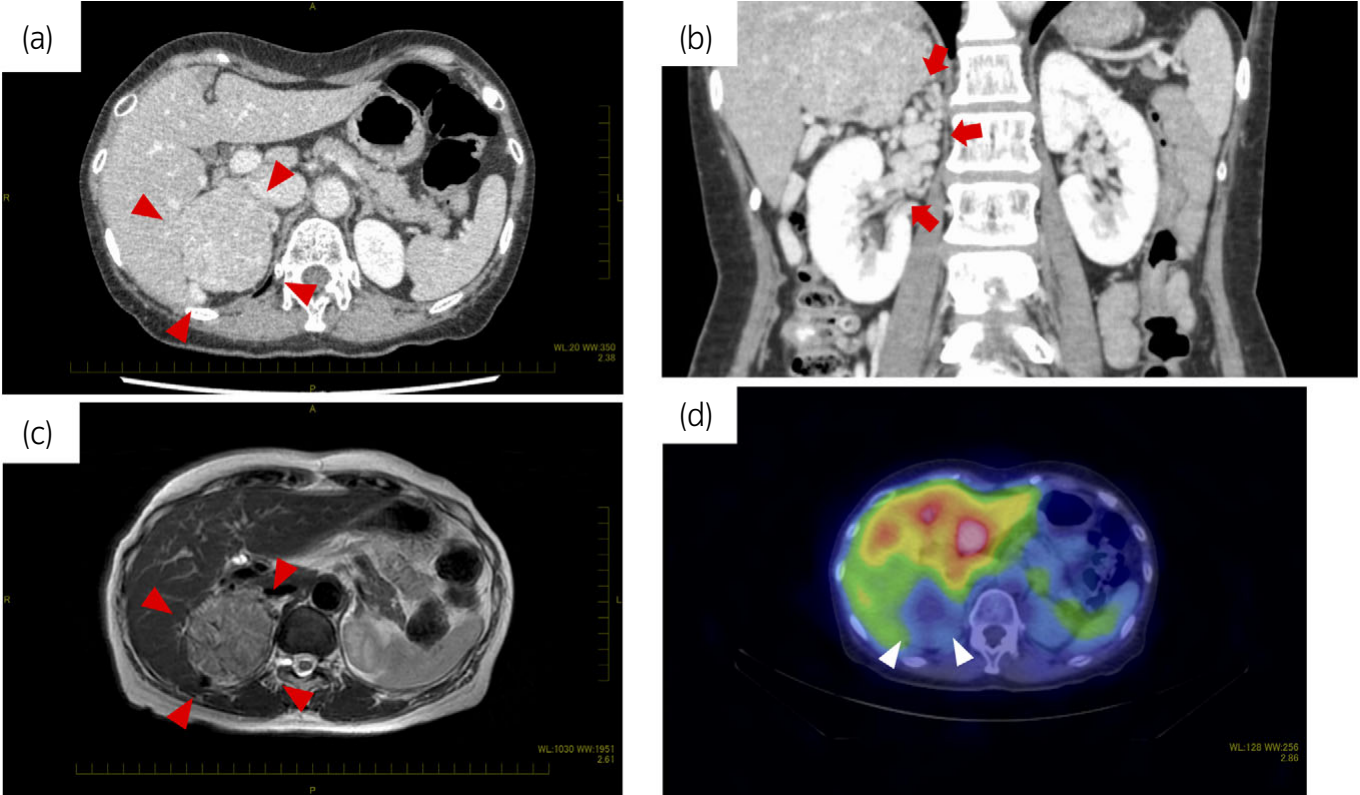
CT scan showed (a) a well‐circumscribed tumor at the superior pole of the right kidney and (b) well‐developed blood vessels around the lesion. (c) The tumor showed a high signal on T2‐weighted MRI, and there was no invasion. (d) ^123^I‐MIBG scintigraphy was negative for radionuclide accumulation in the mass.

A 66‐year‐old woman presented to the local hospital with abdominal pain, and a mass lesion in the right adrenal gland was detected on CT scan. She was referred to our department for further evaluation. She had no medical history other than a thyroid cyst, and no family history of endocrine diseases. Her height and weight were 151 cm and 41 kg, respectively. Blood pressure was normal at around 100/60 mmHg. Her blood and urine tests were normal, and there was no evidence of glucose intolerance or lipid metabolism abnormalities. Endocrinological examinations revealed no abnormalities suggestive of a functional tumor (Table 1). CT showed an adrenal tumor of 66 × 60 × 52 mm (Fig. 1a). The mean CT value of the tumor was about 50 HU, and there were some petechial calcifications inside the tumor. The tumor showed strong contrast enhancement. Well‐developed thick and tortuous blood vessels surrounded the tumor (Fig. 1b). On MRI, the tumor showed a high signal on T2‐weighted images and diffusion‐weighted images (Fig. 1c). The entire tumor was stained early with contrast media. Chemical shift imaging showed no signal change in the tumor between in‐phase and opposed‐phase, indicating that there was no fatty component or hemorrhage inside. The tumor was in contact with the liver, the right kidney, and the inferior vena cava, but no definitive invasion was observed. ^123^I‐MIBG scintigraphy revealed negative radionuclide accumulation in the mass (Fig. 1d).

**Table 1:** Endocrinological examinations

Blood chemistry	
TSH	0.43 μIU/mL (0.45–4.95)
free T3	3.7 pg/mL (2.3–4.3)
free T4	1.47 ng/dL (1.00–1.64)
ACTH	6.3 pg/mL (7.2–63.3)
Cortisol	5.84 μg/dL (7.2–63.3)
PAC	45.6 pg/mL (<120)
ARR	28.5 (<200)
Adrenaline	0.05 ng/mL (0.0–0.17)
Noradrenaline	0.19 ng/mL (0.15–0.57)
Metanephrine	95 pg/mL (<130)
Normetanephrine	98 pg/mL (<506)
24‐hour urine collection	
Adrenaline	14.4 μg/24 h (1.1–22.5)
Noradrenaline	122 μg/24 h (29.2–118)
Metanephrine	0.20 mg/24 h (0.05–0.20)
Normetanephrine	0.26 mg/24 h (0.10–0.28)

The reference range for each parameter is shown in parentheses.

Taking these results together with the characteristics of the tumor including its large size and abundant hypervascularity on CT and MRI, a pheochromocytoma with negative ^123^I‐MIBG scintigraphy was suspected preoperatively. Adrenal carcinoma, oncocytoma, or nonfunctional adrenocortical adenoma could not be ruled out because of the absence of clinical symptoms or endocrine abnormalities, and the large tumor size and hypervascularity. Considering the possibility of malignancy, we decided to perform adrenalectomy.

**Fig. 2:**
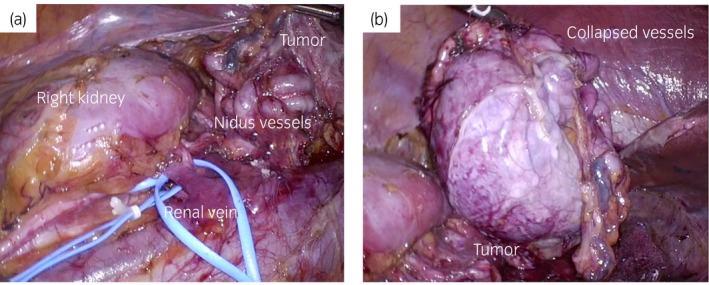
(a) At surgery, the tumor was located at the superior pole of the right kidney and was surrounded by thick, tortuous blood vessels that formed a nidus. In the renal hilum, there was a thick vessel branch from the main renal artery, leading directly to the tumor and the nidus. (b) After stapling the branch, the nidus vessels collapsed. The adrenal gland, tumor, and the growth vessels were all removed together.

The patient underwent transabdominal laparoscopic right adrenalectomy with 5 ports. The port configuration was same as that of right laparoscopic nephrectomy to prepare if a nephrectomy was also needed. After mobilizing the liver, the transverse colon, and the duodenum, the retroperitoneal space was created by lifting the right kidney and the right ureter. The renal veins and the renal artery were identified and secured, then the spaces between the tumor and the liver or the kidney were dissected to gain mobility around the tumor. The tumor was located at the superior pole of the right kidney and was surrounded by thick, tortuous blood vessels that formed a nidus (Fig. 2a). There was no invasion of the tumor into surrounding organs, and adhesions were not prominent. The main drainage veins were supposed to be the ovarian vein and the renal capsular veins because they were developed particularly. We tried leaving the drainage veins open as much as possible so that the venous pressure of the tumor kept low. After dividing some adrenal arteries and dissecting the renal artery to the hilum, we encountered a thick vessel branch from the main renal artery, leading directly to the tumor and the nidus. The nidus was stapled carefully with an endoscopic vascular stapler not to sacrifice the renal branches. We did not use Hem‐o‐lok clips due to the size of the branch. After selective stapling of this feeding branch, the nidus vessels had clearly collapsed. Finally, all the drainage veins were dissected with a vessel sealer. In the end, the adrenal gland, tumor, and growth vessels were all removed together (Fig. 2b). No changes in blood pressure were observed intraoperatively. The operation time was 155 min, and the estimated blood loss was 10 mL.

**Fig. 3:**
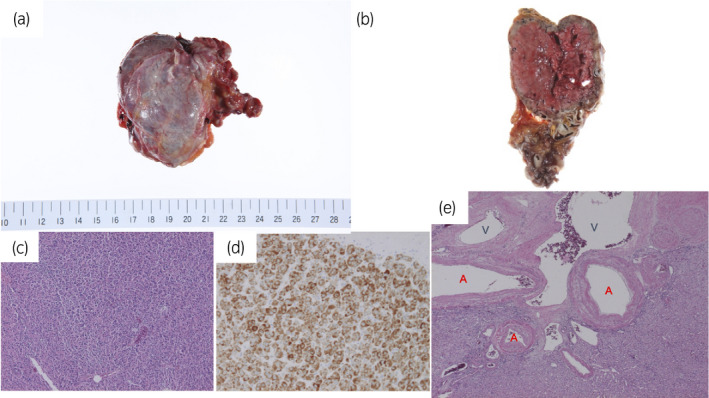
Macroscopically, (a) the tumor was 68 × 48 × 37 mm in diameter and well defined. (b) The cut surface was homogeneous brown in color. Histologically, (c) solid nests with eosinophilic cells (×4, HE stain). (d) Immunohistochemical study was positive for Melan A. (e) The specimen of the nidus vessels revealed both arterial and venous vessels without capillary communications (×2, HE stain, A, arterial; V, venous).

The right adrenal specimen was 96 grams in weight. Macroscopically, the tumor was a well‐defined, nodular lesion in the adrenal gland (Fig. 3a). The cut surface was a homogeneous brown in color (Fig. 3b). Histologically, the nodule consisted of solid or focally tubular epithelium with eosinophilic cytoplasm (Fig. 3c). An immunohistochemical study revealed positive findings for SF1, Melan A (Fig. 3d), synaptophysin, calretinin, and inhibin, and was negative for chromogranin A and SOX10. There was no extracapsular invasion, vascular invasion, or necrosis of the tumor, and mitotic images were inconspicuous, resulting in a modified Weiss score of 2 points. The final pathological diagnosis was adrenocortical adenoma. The nidus vessels were found adjacent to the tumor. Microscopically, both arterial and venous vessels were found in the specimen with no evident of capillary communications (Fig. 3e). The nidus vessels surrounding the tumor were diagnosed as an AVM.

The patient’s postoperative course was uneventful, and she was discharged on the seventh postoperative day. No recurrence or metastasis was observed for 4 months after surgery.

## Discussion

In this case, it was difficult to make a preoperative diagnosis due to the large size of the adenoma and prominent growth of vessels around the tumor. In general, hypervascularization around adrenal tumors is associated with malignancy, pheochromocytoma, and other hyperemic lesions. The well‐developed vessels around the tumor were a nidus of the AVM. Renal AVMs are rare pathological communications between the renal arteries and veins, without interconnecting capillaries. Their prevalence is approximately 0.04% of the general population.^2^ There are two types of AVM, namely the acquired type, caused by renal biopsy, trauma, or partial nephrectomy, and the idiopathic type, which is further classified by angiographic findings into the cirsoid type and the aneurysmal type. The patient was radiographically diagnosed as the cirsoid type, which has tortuous blood vessels and multiple arteriovenous interconnections. For the diagnosis, CT or angiography is useful to visualize the nidus vessels.^3^ Angiography is more efficient for detailed definition of blood vessels. We did not perform angiography before surgery because of the possibility of pheochromocytoma. If angiography had been performed, it might have been useful for the diagnosis of the renal AVM. In addition, selective angiography could have allowed us to evaluate the morphology of the feeding vessels to the tumor before surgery.

Regarding the relationship between the AVM and the adrenal tumor in this case, we hypothesized that the adrenal adenoma preceded the AVM, and the AVM was induced by the hypervascular nature of the tumor, which is often observed in malignant tumors; the AVM preceded the tumor, and the tumor enlarged due to the abnormally increased blood flow; or a small congenital AVM had grown over time, complicated by pressure on the venous drainage caused by development of the large adrenal tumor. Because there are no previous reports on the direct relationship between an adrenal tumor and an AVM, we believe that the adrenal adenoma and the AVM occurred coincidentally. More reports of this rare combination in the future could determine their precise relationship.

## Conclusion

We report a case of a renal AVM surrounding a giant adrenal tumor. To the best of our knowledge, this is the first reported case of a combined giant adrenocortical adenoma and a renal AVM.

## Conflict of interest

The authors declare no conflict of interest.
